# Understanding the quality of life (QOL) issues in survivors of cancer: towards the development of an EORTC QOL cancer survivorship questionnaire

**DOI:** 10.1186/s12955-018-0920-0

**Published:** 2018-06-04

**Authors:** Marieke van Leeuwen, Olga Husson, Paola Alberti, Juan Ignacio Arraras, Olivier L. Chinot, Anna Costantini, Anne-Sophie Darlington, Linda Dirven, Martin Eichler, Eva B. Hammerlid, Bernhard Holzner, Colin D. Johnson, Meropi Kontogianni, Trille Kristina Kjær, Ofir Morag, Sandra Nolte, Andrew Nordin, Andrea Pace, Monica Pinto, Katja Polz, John Ramage, Jaap C. Reijneveld, Samantha Serpentini, Krzysztof A. Tomaszewski, Vassilios Vassiliou, Irma M. Verdonck-de Leeuw, Ingvild Vistad, Teresa E. Young, Neil K. Aaronson, Lonneke V. van de Poll-Franse, on behalf of the EORTC QLG

**Affiliations:** 1 Division of Psychosocial Research & Epidemiology, The Netherlands Cancer Institute, Amsterdam, The Netherlands; 2 Department of Medical Psychology, Radboud University Medical Center, Nijmegen, The Netherlands; 3 Experimental Neurology Unit, School of Medicine and Surgery, University of Milano-Bicocca, Monza, Italy; 4 Milan Center for Neuroscience, Milan, Italy; 5 Oncology Departments, Complejo Hospitalario de Navarra, Pamplona, Spain; 6 Pôle Neurosciences Cliniques, Service de Neuro-Oncologie, Aix-Marseille Université, Marseille, France; 7 Departmental Psychoncology Unit, Sant’Andrea Hospital, Sapienza University of Rome, Rome, Italy; 8 Faculty of Health Sciences, University of Southampton, Southampton, UK; 9 Department of Neurology, Leiden University Medical Center, Leiden, The Netherlands; 10 Department of Neurology, Haaglanden Medical Center, The Hague, the Netherlands; 11 Division of Epidemiology and Health Services Research at Institute of Medical Biostatistics, Epidemiology and Informatics (IMBEI), Mainz, Germany; 12 Department of Otolaryngology Head and Neck Surgery, Sahlgrenska University Hospital, Göteborg, Sweden; 13 Department of Psychiatry and Psychotheraphy, Division of Psychooncology, Innsbruck Medical University, Innsbruck, Austria; 14 University Surgical Unit, University Hospitals Southampton, Southampton, UK; 15 Department of Nutrition & Dietetics, School of Health Sciences and Education, Harokopio University, Athens, Greece; 16 Unit of Survivorship Research, Danish Cancer Society Research Center, Copenhagen, Denmark; 17 Oncology Institute, Chaim Sheba Medical Center, Tel-Hashomer, Israel; 18 Department of Psychosomatic Medicine Center for Internal Medicine and Dermatology, Charité - Universitätsmedizin Berlin, Berlin, Germany; 19 East Kent Gynaecological Oncology Centre, Margate, UK; 20 Neuroncology Unit, National Cancer Institute Regina Elena, Rome, Italy; 21 Rehabilitation Unit, Department of Supportive Care, Istituto Nazionale Tumori – IRCCS- Fondazione G. Pascale, Naples, Italy; 22 Evangelische Kliniken Gelsenkirchen, Gelsenkirchen, Germany; 23 Department of Gastroenterology and Hepatology, Hampshire Hospitals NHS Foundation Trust, Basingstoke, UK; 24 Department of Neurology and Brain Tumor Center Amsterdam, VU University Medical Center, Amsterdam, The Netherlands; 25 Unit of Psychoncology - Breast Unit, Istituto Oncologico Veneto (IOV)-IRCCS, Padua, Italy; 26 Health Outcomes Research Unit, Department of Geriatrics, Gerontology, and Social Work, Faculty of Education, Ignatianum Academy, Krakow, Poland; 27 Department of Radiation Oncology, Bank of Cyprus Oncology Centre, Nicosia, Cyprus; 28 Department of Otolaryngology / Head & Neck Surgery, VU University Medical Center, Amsterdam, The Netherlands; 29 Department of Gynecology and Obstetrics, Sorlandet Hospital Kristiansand, Kristiansand, Norway; 30 Lynda Jackson Macmillan Centre, East & North Hertfordshire NHS Trust including Mount Vernon Cancer Centre, Northwood, UK; 31 Comprehensive Cancer Centre South (CCCS), Eindhoven Cancer Registry, Eindhoven, The Netherlands; 32 Tilburg University, Tilburg, The Netherlands

**Keywords:** Cancer survivor, Disease-free, Health- related quality of life, Survivorship questionnaire, Disease-free, Oncology

## Abstract

**Backround:**

The number of cancer survivors is growing steadily and increasingly, clinical trials are being designed to include long-term follow-up to assess not only survival, but also late effects and health-related quality of life (HRQOL). Therefore it is is essential to develop patient-reported outcome measures (PROMs) that capture the full range of issues relevant to disease-free cancer survivors. The objectives of this project are: 1) to develop a European Organisation for Research and Treatment of Cancer (EORTC) questionnaire that captures the full range of physical, mental and social HRQOL issues relevant to disease-free cancer survivors; and 2) to determine at which minimal time since completion of treatment the questionnaire should be used.

**Methods:**

We reviewed 134 publications on cancer survivorship and interviewed 117 disease-free cancer survivors with 11 different types of cancer across 14 countries in Europe to generate an exhaustive, provisional list of HRQOL issues relevant to cancer survivors. The resulting issue list, the EORTC core questionnaire (QLQ-C30), and site-specific questionnaire modules were completed by a second group of 458 survivors.

**Results:**

We identified 116 generic survivorship issues. These issues covered body image, cognitive functioning, health behaviors, negative and positive outlook, health distress, mental health, fatigue, sleep problems, physical functioning, pain, several physical symptoms, social functioning, and sexual problems. Patients rated most of the acute symptoms of cancer and its treatment (e.g. nausea) as no longer relevant approximately one year after completion of treatment.

**Conclusions:**

Compared to existing cancer survivorship questionnaires, our findings underscore the relevance of assessing issues related to chronic physical side effects of treatment such as neuropathy and joint pain. We will further develop a core survivorship questionnaire and three site-specific modules for disease-free adult cancer survivors who are at least one year post-treatment.

**Electronic supplementary material:**

The online version of this article (10.1186/s12955-018-0920-0) contains supplementary material, which is available to authorized users.

## Background

With continuing improvement in early detection and treatment, and an aging population, the number of cancer survivors is increasing steadily. This has resulted in a growing interest in evaluating the health-related quality of life (HRQOL) of cancer survivors [1]. Increasingly, clinical trials and comparative effectiveness studies are being designed to include long-term follow-up to assess, in addition to survival, late effects of treatment and HRQOL. In order to integrate HRQOL in such studies, it is essential to develop patient-reported outcome measures (PROMs) that capture the full range of issues relevant to disease-free cancer survivors.

Many of the cancer-related HRQOL questionnaires that are available today, including the European Organisation for Research and Treatment of Cancer core questionnaire (EORTC QLQ-C30) [2] and the Functional Assessment of Cancer Therapy – General (FACT-G) [3], with their supplementary site-specific modules, may not be entirely appropriate for assessing the experiences of disease-free cancer survivors. These questionnaires include items assessing acute and treatment-related symptoms (e.g., vomiting) that are typically no longer relevant in the post-treatment survivorship period. Conversely, they may not adequately address physical and psychosocial health problems particularly relevant to cancer survivors (e.g., fear of recurrence, return to work).

Questionnaires that have been developed specifically for use among (long-term) cancer survivors include the Cancer Problems in Living Scale (CPILS) [4, 5], Impact of Cancer (IOC/IOCv2) [6–8], Quality of Life in Adult Cancer Survivors (QLACS) [9, 10], Quality of Life Cancer Survivors (QoL-CS) [11] and Satisfaction with Life Domains Scale for Cancer (SLDS-C) [12]. These questionnaires focus primarily on psychosocial aspects of survivorship and pay relatively little attention to assessing chronic physical effects of cancer and its treatment [13]. Additionally, while they assess generic (e.g., fear of recurrence) HRQOL issues relevant to the survivorship period, these questionnaires do not include condition-specific issues which may persist or arise as a long-term consequence of treatment (e.g., genitourinary problems in prostate cancer survivors or lymphedema problems in breast cancer survivors). Also, they often have been based on investigations of a limited number of cancer sites and on survivors living in the United States [4–8, 12], thus limiting their generalizability to other survivor populations in other countries. Finally, for most of these questionnaires, there is only limited information available about their psychometric properties, or the psychometrics have been based on only a small number of cancer survivor populations [13].

For this reason, the EORTC Quality of Life Group (QLG) has embarked on a project with the primary objective of developing a HRQOL assessment approach that captures the full range of issues relevant to disease-free cancer survivors, both in general and for specific cancer sites. Many definitions of cancer survivorship have been used in the literature [14]. We use the term “cancer survivor” to describe any person who has been diagnosed with cancer who has completed treatment with curative-intent (with the exception of maintenance treatment) and is disease-free (no evidence of active cancer). Since The EORTC QLG’s current portfolio of measures has been primarily designed to assess patients’ HRQOL during treatment and shortly after completion of treatment, it makes sense to begin use of survivorship measures once the acute symptoms of the disease and its treatment have resolved. An important secondary objective of this project is to determine the most appropriate minimum time since end of primary treatment for commencing use of survivorship HRQOL measures.

**Fig. 1:**
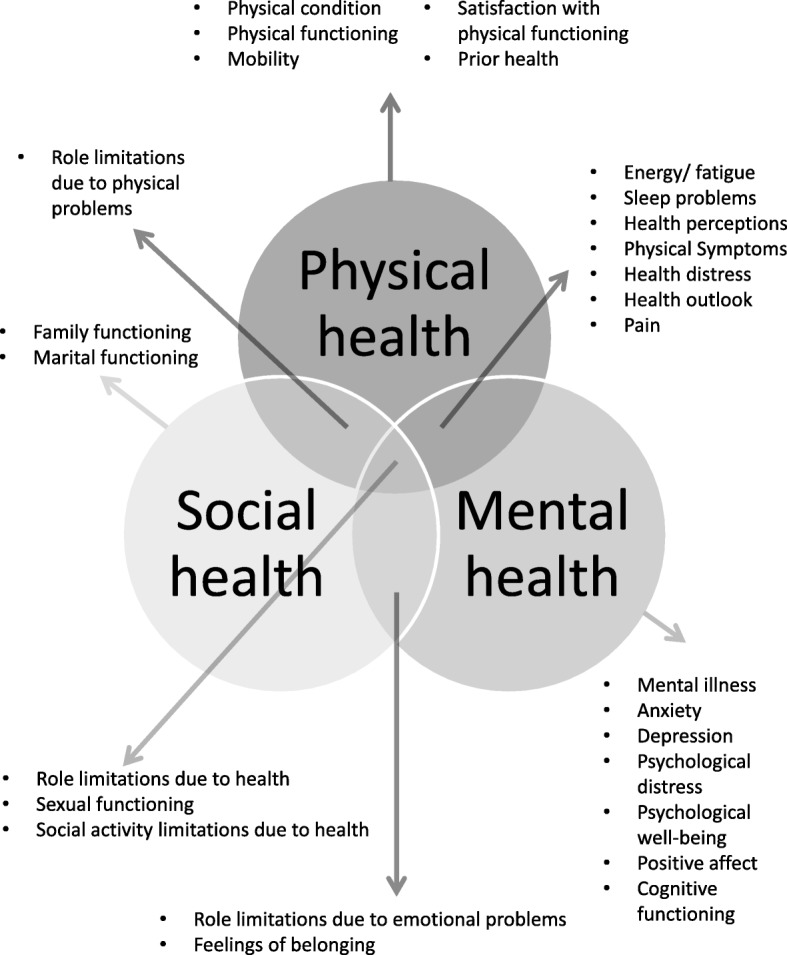
Three-dimensional theoretical framework of health. In this framework health is assessed by multiple health indicators

The conceptual framework we employed for the development of the questionnaire followed the World Health Organization (WHO) definition of health, dating from 1948, as “a state of complete physical, mental, and social well-being and not merely the absence of disease or infirmity”. Combined with the Medical Outcomes study (MOS) framework it delineates three key dimensions of health: physical, mental and social [15]. These three dimensions can be assessed by several types of indicators (see Fig. 1). Some of these indicators reflect primarily one of the three dimensions (e.g., physical functioning) and others reflect two or three dimensions (e.g., fatigue) [15].

In terms of measurement strategy, this project follows the tradition within the EORTC QLG of involving patients from a range of countries, cultures and languages at every step in the developmental process. Additionally, given the goal of addressing both generic and cancer site-specific survivorship issues, the project encompasses a broad range of diagnostic groups. We follow the EORTC QLG’s four- phase process of questionnaire development [16]: 1) generation of relevant HRQOL of issues; 2) conversion of HRQOL issues into a set of questionnaire items; 3) questionnaire pre-testing; and 4) large-scale international field testing. In the current paper we report on the results of the first phase of this project.

## Methods

**Fig. 2:**
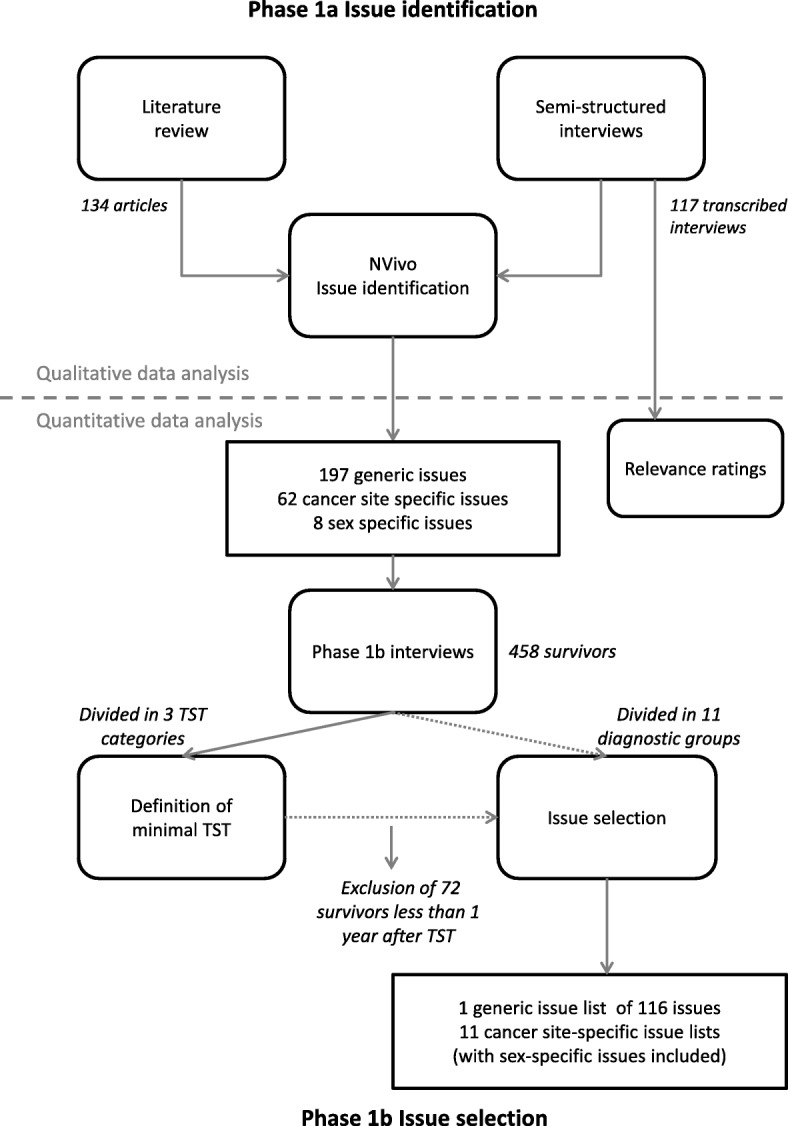
Work flow of phase I. TST time since completing last treatment

Phase I had two aims: 1) to determine the full range of issues relevant to disease-free cancer survivors, both in general and for specific cancer sites; and 2) to determine the most appropriate minimum time since end of primary treatment for commencing use of survivorship HRQOL measures. It consisted of two sub-phases: In phase 1a we generated an exhaustive list of HRQOL issues, drawing upon two primary sources: the literature and cancer survivors. In phase 1b we asked a sample of cancer survivors to rate the QLQ-C30 to determine at which time after treatment completion the acute symptoms and side-effects related to cancer and its treatment are no longer relevant. We considered the diminishing prevalence of these symptoms an indicative of the need to shift to the assessment of longer term survivorship issues. In addition, the survivors participating in phase 1b rated the list of HRQOL issues developed in phase 1a to identify the issues relevant to disease free-survivors. In this phase only quantitative data analyses were applied. The workflow of the study is also presented in Fig. 2.

### Literature search

In October 2014, we performed a literature search in PubMed and PsycINFO, the goal of which was to identify the full range of HRQOL issues relevant to *all* adult disease-free cancer survivors, irrespective of their specific diagnosis. We used the following search terms: (“Survivors”[Major] OR “Survivors/psychology”[Major]) AND (“neoplasms”[Major] OR “Carcinoma”[Major]) AND (“Quality of Life”[Mesh] OR “patient-reported outcomes” OR “health-related quality of life” OR “wellbeing” OR “well-being” OR “Mental Health”[Major] OR “Physical Fitness/psychology”[Major] OR “Physical Fitness/physiology”[Major] OR “Health Status”[Major] OR “late effects”) AND adults. We included only original articles published in English that investigated HRQOL in adult, disease-free cancer survivors. Articles reporting only scale scores without reference to specific survivorship issues were excluded. Two of the authors (MVL and OH) screened references independently, and disagreements were resolved by consensus.

### Study sample

For phase 1a & b we recruited cancer survivors from hospitals from four geographic regions: the United Kingdom, Northern Europe (Denmark, Germany, the Netherlands, Norway, Sweden), Southern Europe (Cyprus, France, Greece, Israel, Italy, Spain), and Central Europe (Austria, Poland). Inclusion of participants for phase 1b took place after completion of phase 1a. Eligible patients were those aged 18 years or older at the time of diagnosis who had sufficient command of their native language and did not have severe psychological or cognitive problems.

To ensure that the survivorship questionnaire would be generic in nature, we recruited survivors with a range of cancer diagnoses, selected on the basis of their prevalence and/or survival rates. This included 11 diagnoses: breast, colorectal, prostate, bladder, gynecological (ovarian, cervix and endometrial), head and neck, lung, and testicular cancer, lymphoma, melanoma, and glioma. Eligible patients had completed their treatment with curative intent (both primary treatment and treatment of recurrent disease) at least 6 months earlier and were disease-free (no evidence of disease). They could be receiving maintenance therapies (e.g., hormonal treatment for primary breast cancer). Although low-grade glioma patients are not treated with curative intent and are not disease-free, they were included in the study because they have a median survival of between 4.7 and 9.8 years [17]. We employed purposive sampling to ensure an approximately equal distribution of patients across diagnoses and time since treatment (see below).

Basic sociodemographic data collected at study entry included: age, sex, education, employment status, and living arrangement. Clinical data collected included primary diagnosis, stage of disease, type of treatment, date of diagnosis, date of start of primary treatment, date of completing primary treatment, recurrences, date of completion of treatment for last recurrence, and comorbidity using the Charlson Index [18].

### Phase 1a survivor interviews: issue generation

We conducted semi-structured interviews with an initial sample of survivors in order to generate an exhaustive list of relevant HRQOL issues. The goal was to complete 10 interviews per diagnostic group, equally distributed over the four geographical regions [16]. First, the respondents were asked open-ended questions about their survivorship experience. Subsequently, respondents were shown the EORTC core questionnaire (QLQ-C30) and, if available, the relevant site-specific questionnaire module [19–32]. These cancer site-specific modules assess the HRQOL issues most relevant to each of the specific patient populations. These modules range in length from 13 to 35 items. Instead of completing these questionnaires, the respondents were asked to rate the relevance of the items on a 4-point scale (not at all, a little bit, quite a bit, or very relevant). The respondents were also asked to identify survivorship issues that they believed to be important that were not included in the QLQ-C30 and, where relevant, site-specific module.

#### Relevance ratings of the EORTC core questionnaire (QLQ-C30)

We evaluated the relevance ratings by composing scales that were in accordance with the QLQ-C30 scale structure: 5 multi-item functioning scales (physical, role, emotional, cognitive, and social functioning), three multi-item symptom scales (fatigue, pain, nausea/vomiting), and 6 single items (dyspnea, insomnia, appetite loss, constipation, diarrhea, and financial difficulties). All scores were linearly transformed to a 0 to 100-points scale. A higher score on a scale means that the survivors considered the items of this particular scale more relevant. We compared the following survivor groups: 0.5 to 2 years since diagnosis, 2 to 5 years since diagnosis, and 5 years or more since diagnosis.

### Issue extraction from literature and interviews

Phase 1a interviews were transcribed and translated in English by the interviewers. We employed thematic analysis [33] using NVivo 10 [34], a software program for qualitative data analyses, to extract a list of relevant survivorship issues from the articles included in the literature review and from the transcribed semi-structured phase 1a interviews. Literature and interviews were analyzed simultaneously using the coding system that evolved during the thematic analyses. Items of the existing questionnaires and the issues described in the qualitative studies were coded in issues that were organized into hierarchical trees. Cancer site or sex-specific issues were extracted in separate coding trees, and kept separately to avoid survivors in phase 1b having to rate too many issues. All issues were consolidated into a provisional list which included generic, site-specific, and sex-specific issues to be completed by a second group of survivors in phase 1b.

### Phase 1b survivor interviews: defining minimal time since treatment completion and issue selection

In the second round of interviews, our goal was to recruit 330 survivors: 10 interviews per survivor group * 11 diagnostic groups * 3 time periods (6 months – 2 years / 2–5 years / > 5 years since completing primary treatment) [16]. The sample was also stratified according to geographical region. The respondents were asked to complete the provisional issue lists, using a 4-point response scale (not at all, a little bit, quite a bit, or very much) to indicate the extent to which they had experienced each issue. The provisional issue lists consisted of a generic survivor issue list, and the sex- and cancer site-specific issues. In addition, they were also asked to complete the QLQ-C30 and, if available, the relevant site-specific module using the same response scale. This was followed by a debriefing interview about any relevant issues missing from the provisional issue lists.

#### Definition of the minimal time since end of treatment for assessing survivorship issues

To determine the minimum time since end of treatment for which the survivorship questionnaire would be relevant, we divided the Phase 1b sample into three time-since-completion-of-last-treatment (TST) groups: (1) 0.5 to 1 years; (2) 1 to 2 years; and (3) 2 years or more since treatment completion. For each TST group we investigated which items of the QLQ-C30 were rated as relevant for that specific group: an item was considered relevant if at least 30% of the respondents in a group endorsed an item (i.e., had experienced the issue at least “a little bit”). We were particularly interested in comparing responses to the QLQ-C30 items between the three TST groups, as the QLQ-C30 contains a number of acute symptom and side-effect items. Our objective here was to determine at what point in time these relatively acute issues were no longer relevant for the majority of respondents, and thus it would be appropriate to begin using survivorship measures.

#### Criteria for issue selection in phase 1b

In this phase, the provisional issue list was reviewed to generate one comprehensive list of generic issues that was relevant to all groups of cancer survivors, regardless of specific diagnosis. In addition, site-specific issue lists and sex-specific issues were generated. As indicated above, we coded an issue as being endorsed by a respondent if it was scored “a little bit” or higher. An issue needed to be endorsed by at least 30% of the survivors in any given diagnostic group to be regarded as an issue relevant to that group. If an issue was endorsed by survivors from 6 or more diagnostic groups, it was then considered to be a generic survivorship issue; otherwise it was deemed to be a cancer-site specific issue. This resulted in 12 consolidated survivorship issue lists: one generic list and 11 cancer-site specific lists.

#### Sub-analyses in the younger age groups

Previous research has shown that younger adult survivors can be particularly impacted by the cancer experience [35–41]. For this reason, we performed post-hoc, age-related subgroup analyses to identify issues that are particularly relevant to survivors younger than 50 years. We divided the total sample into five age groups (< 40; 40–50; 50–60; 60–70; 70+ years). Issues endorsed as relevant by at least 30% of respondents under the age of 50 years that would otherwise have been excluded on the basis of ratings by respondents above the age of 50 (i.e. low endorsement in the older sample causing the endorsement in the total sample to be below 30%) were retained in the generic list as being particularly relevant for younger cancer survivors. Our expectation was that we would retain issues likely to be specifically relevant to younger cancer survivors such as problems in obtaining a mortgage or family planning.

## Results

### Literature review

**Fig. 3:**
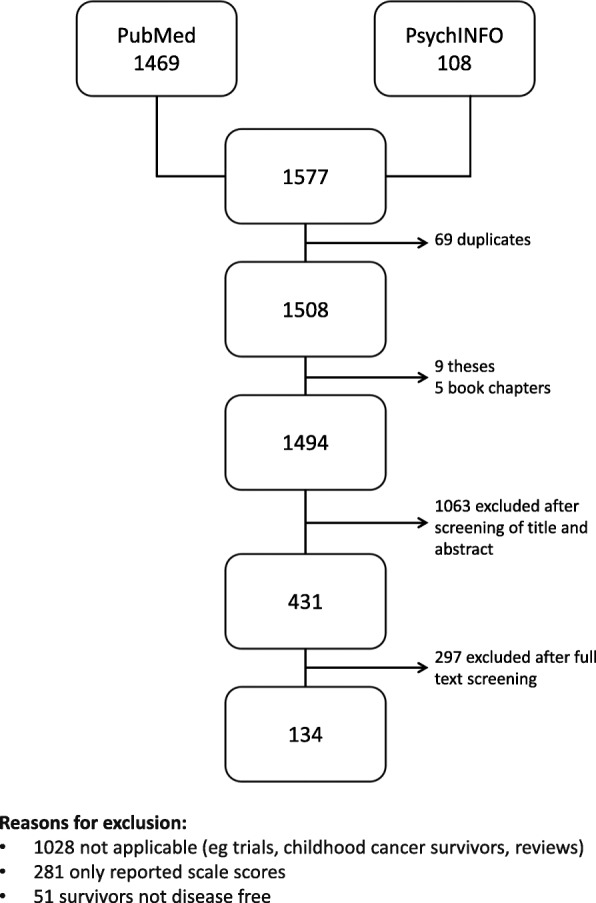
Prisma flow chart of the literature review

The literature search identified 1494 publications, of which 134 were retained for issue extraction (for details see Fig. 3). The list of 134 articles included in the review can be found in Additional file 1. Research articles included in the review were most commonly qualitative studies, studies developing cancer survivor specific measures, or studies reporting the use of a self-constructed study-specific measure.

### Semi-structured interviews phase 1a: issue generation

For phase 1a, 117 survivors were interviewed between August 2014 and May 2015 in nine different European countries (Table 1). The average age of the survivors was 57 years (SD = 13.6 years), and 54% had received their cancer diagnosis between 2 and 5 years ago.

**Table 1:** Number of cancer survivors per cancer site and per region included in phase 1a and 1b

	Phase 1a	Phase 1b
Cancer site		
Bladder	7	32
Breast	17	53
Colorectal	12	46
Glioma	10	36
Gynecological	12	49
Head & neck	10	44
Lung	8	41
Lymphoma	9	38
Melanoma	11	38
Prostate	11	44
Testicular	10	37
Total	117	458
Region		
Northern Europe	30	147
Southern Europe	46	126
English speaking	13	98
Central Europe	28	87
Time since diagnosis		
0.5 to 2 years	21	111
2 to 5 years	63	189
more than 5 years	33	158
Time since completing last treatment^a^		
0.5 to 1 year	–	72
1 to 2 years	–	105
2 to 5 years	–	172
more than 5 years	–	109

For phase 1b only, also the number of survivors per time since completion of last treatment category is reported

^a^For phase 1a only information was available regarding date of diagnosis and date of recurrence

### Issue extraction from literature and interviews

In the first step, we identified 1555 issues from the 117 interviews and the first 75% of the research articles. These issues were classified into 11 themes: mental health, physical symptoms, cognitive changes, role functioning (including work), meaning of cancer, health behaviors, spirituality, social functioning (including feelings of belonging), financial issues, body image, and sexuality. In the next step we reduced this list to 718 issues by combining issues that were very similar or formed a continuous scale (e.g. “depression” and “feeling depressed” were combined into “feeling depressed”). The remaining 25% of studies was coded using this 718 issues coding system. We did not identify new issues in these studies. In the last step, the total number of issues was further reduced. Issues that were very specific were combined. For example, “fear of recurrence when having physical symptoms” and “fear of recurrence around physical exams” were combined into “fear of recurrence”. Issues reflecting states like “being retired” were not included, as they cannot be assessed on a 4-point scale, and would not be informative for an assessment of HRQOL. Issues stating a *change* in physical symptoms were not included. General issues like “emotional problems” were not included as we believed that they were better captured by more informative issues, for example, “being worried”, “fear of dying”, “anxiousness”, “feeling stressed”, “feeling depressed”. This resulted in 197 generic, 62 cancer site-specific (e.g. pain during urination), and 8 sex-specific (e.g. feeling less feminine) issues.

#### Relevance ratings of the EORTC core questionnaire (QLQ-C30)

**Fig. 4:**
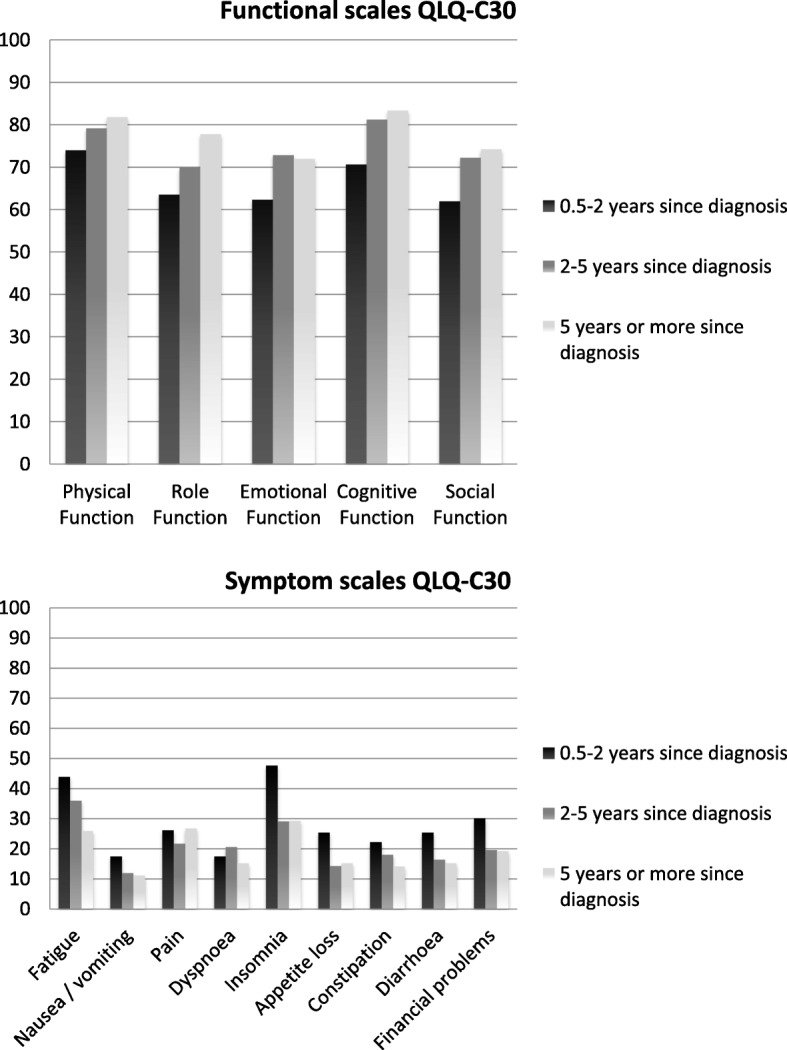
Relevance ratings of the functional and symptom scales of the QLQ-C30 per time since diagnosis group. The y-axis shows the relevance ratings of the QLQ-C30. A higher score on a scale means that the survivors considered the items of a particular scale more relevant

The relevance ratings are presented in Fig. 4. The figure shows that the functioning scales were still considered relevant by the survivors, with the perceived relevance increasing with longer time since diagnosis. A number of the symptom items and scales were considered less relevant, especially when more time had passed since diagnosis. Two years after diagnosis, nausea/ vomiting, appetite loss, constipation, and diarrhoea were seldom rated as being relevant. The ratings showed that particularly insomnia was considered highly relevant by the survivors less than 2 years after diagnosis and fatigue by the survivors who were less than 5 years since diagnosis.

### Phase 1b interviews: defining minimal time since treatment completion and issue selection

Between November 2015 and August 2016, we interviewed 458 survivors from 23 centers in 14 countries for phase 1b (Table 1). The mean age of the sample was 59 years, and 46% was female. Sixty percent had been diagnosed with stage I or II cancer, and 16% had experienced disease recurrence in the past. The average time since last treatment was 3.6 years (Table 2).

**Table 2:** Basic demographics, disease and treatment characteristics of the survivors included in phase 1b

Survivors	Phase 1a	Phase 1b	
	*N* = 117	Total *N* = 458	Subsample *N* = 386^a^
Age			
Mean ± SD (years)	57 (13.6)	59 (13.8)	59 (13.7)
Sex (%)			
male	58 (50%)	246 (54%)	207 (54%)
Partner status^b^			
in relationship	90	367	313
widower/ divorced/ separated	9	61	49
single	14	50	43
Education (%)			
none or primary school only	15 (13%)	59 (13%)	47 (12%)
high school	46 (39%)	167 (36%)	140 (36%)
college or university	53 (45%)	222 (48%)	190 (49%)
missing	3 (3%)	10 (2%)	9 (2%)
Work status (%)			
working	59 (50%)	205 (45%)	180 (47%)
retired	43 (37%)	185 (40%)	157 (41%)
unemployed	4 (3%)	24 (5%)	17 (4%)
homemaker	4 (3%)	22 (5%)	17 (4%)
disabled	5 (4%)	10 (2%)	7 (2%)
other or missing	2 (2%)	12 (2%)	10 (3%)
Disease recurrence (%)	11 (9%)	75 (16%)	66 (17%)
Tumor stage^c^ (%)			
stage I	19 (16%)	101 (23%)	88 (24%)
stage II	45 (38%)	131 (30%)	108 (28%)
stage III	29 (25%)	113 (25%)	95 (25%)
stage IV	3 (3%)	40 (9%)	34 (9%)
stage unknown	13 (11%)	47 (11%)	40 (10%)
no stage determined	8 (7%)		
Time since completing primary treatment			
Mean (SD) (years)	–	4.2 (4.0)	4.8 (4.0)
Time since completing last treatment			
Mean (SD) (years)	–	3.6 (3.2)	4.4 (3.2)
Therapy^b^			
surgery	98	342	326
chemotherapy	77	254	222
radiotherapy	54	238	209
hormonal therapy	15	49	48
monoclonal antibodies	2	21	18
cell transplantation		6	6
active surveillance	7	66	59
current maintenance therapy	8	61	47

Percentages are given in the cases that categories are mutually exclusive

*N* number, *SD* Standard deviation

^a^subsample of phase 1b that consists of the survivors who are at least 1 year after treatment completion

^b^categories are not mutual exclusive, e.g. one can be a widower and have a new relationship

^c^for glioma survivors in Phase 1b tumor grading was used, we included per tumor grade: grade 1: 2 survivors; grade 2: 8 survivors; grade 3: 14 survivors; grade 4: 1 survivor

**Fig. 5:**
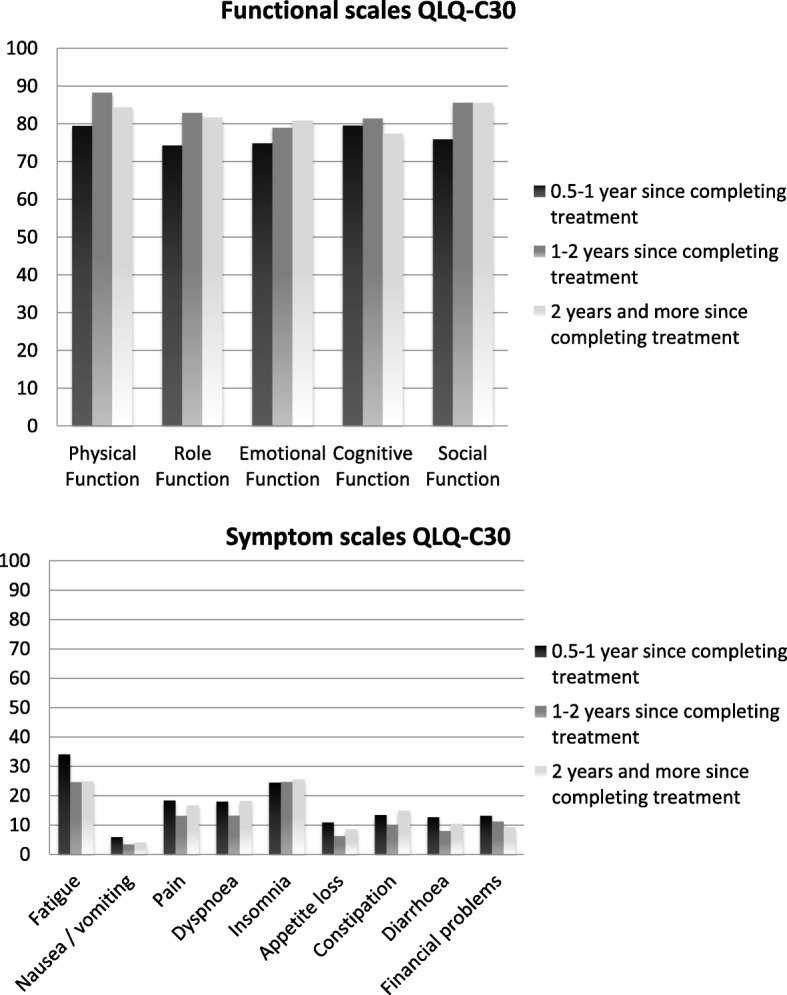
Functional and symptom scales of the QLQ-C30 per time since last treatment category. The y-axis shows the scores on QLQ-C30. On the functional scales a higher score represents a better level of functioning and on the symptom scales a higher score represents a higher level of symptoms

We started our analyses by defining the post-treatment survivorship period in the complete phase 1b sample by comparing the three subgroups (0.5–1 year, 1 to 2 years, and 2 years or more post-treatment). The following QLQ-C30 items were rated as being relevant in the 0.5–1 year post-treatment survivors subgroup, but no longer so in the other two TST subgroups: needing to stay in bed or a chair during the day, pain interfering with daily activities, physical condition or treatment interfering with family life and social activities, and physical condition or treatment causing financial difficulties. Scores on the functional and symptom scales of the QLQ-C30 showed an increase in physical, role emotional and social functioning one year after completion of treatment, and a decrease in fatigue (Fig. 5). After this first year these scores tended to stabilize. Based on these findings we decided to employ the one year post-treatment mark as the threshold for recommending transitioning to the use of survivorship measures.

Additionally, the analyses of the QLQ-C30 data showed that the following items were of low relevance to *all* of the TST survivor subgroups: “Do you have any trouble taking a short walk outside of the house?”, “Do you need help with eating, dressing, washing yourself or using the toilet?”, and those items assessing appetite, nausea, vomiting, and gastrointestinal symptoms.

For the subsequent selection of issues to be included in the survivorship questionnaire, only the 386 respondents who were at least 1 year post-treatment were included. Table 3 displays the 116 issues that were reported as being relevant by respondents in at least 6 of the diagnostic groups. Thirty-four percent of these consisted of issues covering physical functioning (e.g. pain, neuropathy, muscle cramp), 32% were mental functioning issues (e.g. body image, anxiety, positive affect), 19% issues were related to social and role functioning (e.g. sexual problems, feelings of belonging) and 16% involved general health perceptions (e.g. negative health outlook and health behavior). Of the 116 issues, 106 were not included in the QLQ-C30. Table 3 shows which issues are overlapping with the QLQ-C30, which were identified from the literature, and which from the interviews.

**Table 3:** Consolidated issue list with generic survivorship issues

Body Image	
• feeling unattractive ^b, c^ • feeling old^b^ • feeling satisfied with your physical appearance^b, c^ • feeling you could not trust your body^b, c^	
Cognitive functioning	
• difficulties with concentration^a,b, c^ • forgetfulness^b, c^ • memory problems^a,b, c^ • problems with multi-tasking^b, c^ • difficulty gathering your thoughts (together)^b, c^ • ability to think (to process information) has slowed down^b, c^	
Health behaviors	
• being alert for symptoms that may signal a return of my cancer^b, c^ • going quickly to my GP due to having (had) cancer^c^ • drinking less alcohol due to having (had) cancer^b, c^ • listening to my body due to having (had) cancer^b, c^ • eating healthily due to having (had) cancer^b, c^ • avoiding the sun or protecting my skin due to having (had) cancer^b, c^ • exercising (more) due to having (had) cancer^b, c^ • avoiding stress in my life due to having (had) cancer^b, c^ • cutting down smoking due to having (had) cancer (not applicable option)^b, c^ • taking better care of yourself due to having (had) cancer^b, c^	
Meaning of cancer	
• other issues not related to cancer bother me more than having had cancer^b, c^ • cancer is a learning experience^b, c^ • having (had) cancer has made me accept my own mortality^b, c^ • overall quality of life^a, b, c^ • being (more) emotional due to having (had) cancer^b, c^ • seeking a deeper meaning in having (had) cancer^b^	
*Negative outlook*	
• concerned with long term effects of cancer treatment^b, c^ • feeling that my life has been suspended because of having (had) cancer^b, c^ • difficulties adapting my life to the physical consequences of having had cancer^b, c^ • still feeling like a cancer patient^b, c^ • experiencing uncertainty about the future^b, c^	
*Positive outlook*	
• appreciating life (more) due to having (had) cancer^b, c^ • being psychologically strong(er) due to having (had) cancer^b, c^ • my personality has changed for the better due to having (had) cancer ^b, c^ • having (had) cancer has given me a purpose in life^b^ • because of having (had) cancer I have reconsidered my priorities in life^b, c^ • standing up for myself (more) due to having (had) cancer^b, c^ • having (had) cancer has given me a reason to make changes in my life^b, c^ • willing to help others (more) due to having (had) cancer^b, c^ • due to having (had) cancer, being (more) understanding of what other people feel^b, c^	
Mental health	
*Depression/behavioral-emotional control*	*Health distress*
• feeling depressed^a, b, c^ • feeling angry or frustrated^b, c^ • feeling stressed^b, c^ • mood swings^b, c^ • needing psychological support^b, c^ • feeling irritable^a, b, c^ • feeling upset about having (had) cancer^b, c^	• fear of recurrence or spread cancer^b, c^ • worried about health^b, c^ • fear of dying^b, c^ • fear of new cancer^b, c^ • fear family members will develop cancer^b, c^
*Anxiety*	
• being worried^a, b, c^ • feeling anxious ^b, c^	
Physical symptoms	
• altered hair structure^b, c^ • weight gain^b, c^ • feeling ill or unwell^b^ • acid reflux^b^ • overall health^a, b, c^	
*Fatigue*	*Sleep problems*
• feeling constantly tired^b, c^ • needing more sleep to function^c^ • feeling exhausted^b, c^ • feeling tired^a, b, c^ • needing time to recover from normal activities^c^ • feeling weak^a, b, c^ • needing to take naps^b^ • sudden attacks of tiredness^b, c^	• problems falling asleep^b, c^ • waking up frequently at night^b, c^ • trouble sleeping^a,b, c^ • waking up too early^b^
*Physical functioning/ mobility*	*Leg problems*
• difficulty carrying something in both hands while climbing stairs^b^ • difficulty taking a long walk^a,b, c^ • difficulty running fast^b, c^ • difficulty carrying something weighing 5 kg^b, c^ • difficulty hiking for 3 km^b, c^ • difficulty walking up a flight of stairs^b, c^ • difficulty doing strenuous activities like carrying a heavy shopping bag or a suitcase^a, b^	• difficulty standing for a long time^b, c^ • restless legs^c^ • swollen feet or legs^b, c^
*Pain*	*Skin Problems*
• headaches^b, c^ • joint pain^c^ • muscle pain^b, c^	• dry and or scaly skin^b, c^ • thin skin^b, c^
*Raynaud*	*Neuropathy*
• hands and/or feet sensitive to hot and cold^c^ • cold or pale fingers or toes^b, c^	• tingling in hands and/or feet^b, c^
*Muscle problems*	*Temperature*
• muscle cramps^b, c^ • muscle weakness^S^	• night sweats^b, c^ • hot flushes^b, c^ • feeling cold^b, c^
Role functioning	
• limited in recreational activities^a,b, c^	
*Work*	
• difficulties returning to work since having (had) cancer^b, c^ • having (had) cancer decreased work performance^b, c^ • career interrupted due to cancer^b, c^	
Feelings of belonging	
*Positive impact*	*Negative impact*
• family relationships are close(r) due to having (had) cancer^b, c^ • having (had) cancer has a positive impact on the relationship with my partner^b, c^ • feeling close(r) to friends since having (had) cancer^b, c^ • friends and family are (more) important since having (had) cancer^b, c^	• feeling that others do not understand the impact of having (had) cancer^b, c^ • not wanting to burden family members^b, c^ • worried about the impact of my cancer on my children^b, c^
Sexual problems	
• feeling guilty for not fulfilling sexual needs of partner^b^	
*Sex-specific sexual problems*	*Sexual frequency*
• vaginal dryness^b, c^ • problems getting or maintaining erection^b, c^	• low interest in sex^b, c^ • sexually active with or without intercourse^b, c^ • avoiding sex^b^
*Sexual pleasure*	
• difficulty becoming sexually aroused^b^ • feeling uneasy with sex^b^ • problems enjoying sex^b^ • problems having an orgasm^b^ • problems with sexual intimacy^b, c^	
Sub-analyses: issues relevant to younger cancer survivors	
• upset with appearance of scar^b, c^ • feeling angry towards body^b, c^ • feeling embarrassed about body^b, c^ • concerned about the ability to have children^b, c^ • having problems with people treating me differently because I have (had) cancer^b, c^ • difficulties talking about cancer ^b, c^ • my personality has changed for the worse due to having (had) cancer^b, c^ • having (had) cancer has made me lose my future life plans or goals^b^ • financial problems caused by problems with getting a loan, mortgage, or insurance^b, c^	

Issues in this list were endorsed by 30% of the survivors in at least 6 of the included cancer sites

^a^issues which are also included in the QLQ-C30

^b^issues identified in the literature

^c^issues identified in the survivor interviews

In addition to generic issues, we also identified survivorship issues that were cancer site-specific. On average, 26 (range 7–48) issues were considered as cancer-site specific per diagnostic group. We intend to use these issues for the future development of cancer site-specific survivorship modules. Among these cancer site-specific survivorship issues, we observed the following trends: body image issues were frequently endorsed by bladder, breast, colorectal, and head & neck cancer survivors. Cognitive functioning problems were rated as highly relevant by glioma, lymphoma, lung, bladder, breast, and head and & neck cancer survivors. Lung cancer and glioma survivors reported having a negative health outlook as highly relevant. Bladder and head & neck cancer survivors frequently endorsed role functioning issues. Lung, lymphoma, and colorectal cancer survivors more often endorsed work-related issues than the other survivor groups. Glioma and lymphoma survivors more frequently rated issues related to a negative impact on feelings of belonging as relevant.

Based on the 94 survivors below the age of 50 years, we identified 10 issues that were relevant for younger survivors and for which relevance declined with age (see Table 3). Three issues were related to body image; the others were related to the ability to have children, being treated differently by people because of having had cancer, difficulties talking about cancer, negative personality change, needing psychological support, loss of future life plans, and financial problems.

## Discussion

In this first phase of our cancer survivorship questionnaire development project, we identified 116 generic survivorship issues. Additionally, on average, we identified 26 site-specific survivorship issues per tumor site, which only partially overlapped with the existing EORTC site-specific modules. We also observed that, approximately one year following completion of cancer treatment, most of the acute disease- and treatment-related symptoms have resolved themselves in the large majority of survivors.

Based on these findings, we will move forward with the development of a core survivorship questionnaire for disease-free adult survivors who are at least one year post-treatment. This questionnaire will retain many of the original items and scales from the QLQ-C30, deleting only those items that assess acute symptoms (nausea/vomiting, appetite loss, constipation, and diarrhea). Additional survivorship issues will be added to expand the scope of issues addressed by the questionnaire. To improve the measurement precision of some of the existing scales of the QLQ-C30, we will collaborate with the EORTC CAT team [42] to select the issues of the generic issue list and the items of the EORTC QLG item library assessing these issues. This survivorship questionnaire can be complemented by cancer-site specific survivorship modules based on and adapted from the existing EORTC cancer site-specific modules.

The period after treatment completion is often described by survivors as more difficult than the treatment itself [43]. The end of the phase of transition from being a patient to resuming normal life [44] can be very positive, but also brings with it feelings of uncertainty about the future and fear of cancer recurrence. During this early survivorship period, patients often begin to process the emotions related to the diagnosis, to find meaning in their experience of having had cancer, and to deal with the lingering effects of treatment. The end of this turbulent immediate post-treatment period appears to represent an appropriate starting point for assessing survivorship issues, as both physical and psychosocial health begin to stabilize. This was corroborated by the increase in physical, role, and social functioning and the decline in fatigue observed in our study sample after the first post-treatment year. We did not observe a further decline in the acute symptoms of cancer treatment, as the prevalence of acute symptoms was already low a half year after treatment completion. The chronic side effects of treatment (pain, dyspnea, insomnia, and fatigue) continued to be relevant for all survivor groups into the longer post-treatment phase.

In accordance with the existing cancer survivorship questionnaires [4–12] our results indicate that feelings of uncertainty about the future, fears related to recurrence of cancer, fears and worries concerning family members, feelings of depression and anger, feelings that others do not understand the impact of cancer, positive impact on social relationships, positive changes in (perception of) life, negative body image, cognitive problems, fatigue, sleeping problems, pain, sexual problems, and dealing with the chronic physical consequences of cancer are all relevant issues for cancer survivors. However, our results also indicate that other issues often included in survivorship questionnaires may be less relevant when rated by a wider range of cancer survivors in an international context. This includes issues related to feelings of guilt, fears related to starting new (romantic) relationships, and feelings of pride about having survived cancer. Also, compared to existing questionnaires, our findings underscore the relevance of assessing issues related to chronic side effects of treatment such as neuropathy and joint pain [4–12]. Over 30% of the issues were related to physical functioning, including chronic physical effects of cancer and its treatment, like Raynaud symptoms, neuropathy, joint pain, and muscle cramps. These issues receive relatively little attention in the existing cancer survivorship questionnaires. The differences between our findings and the existing survivorship questionnaires may reflect both culture (including differences between the American and European health care systems) and the fact that we included a wider range of diagnostic groups in our study, and placed relatively less emphasis on breast cancer and non-solid cancer survivors, as has typified the developmental phase of other survivorship questionnaires.

Another important finding from our study is that there is not only a fairly large number of condition-specific physical health issues in cancer survivorship, but also differences in the extent to which various psychosocial issues are perceived as relevant by specific cancer diagnostic groups. These differences in perceived relevance of survivorship issues may reflect differences in survival rates between the cancer types, the average age at diagnosis, the nature of the chronic side effects of the various treatments, and whether a cancer diagnosis is sex-specific. For example, glioma and lung cancer survivors reported issues related to the negative impact of cancer on their lives and issues related to struggles with family and friends as being very relevant; lung, lymphoma, and colorectal cancer survivors more frequently rated work-related issues; and bladder and head & neck cancer survivors more frequently endorsed role functioning issues. Most of these site-specific survivorship issues are currently not included in the existing site-specific modules of the EORTC.

The literature consistently shows that younger cancer survivors report a higher impact of their cancer experience on HRQOL [45, 46], including higher levels of distress, than older cancer survivors. This is likely related to the fact that relatively younger survivors are confronted with a life-threatening illness at a time when many are in the midst of forming relationship bonds, starting and raising families, and trying to establish a workable balance between career and family life. During this period of young adulthood, a serious illness such as cancer is less expected, and may therefore be more disruptive. Also, younger cancer patients and survivors may perceive themselves as having more to lose in terms of future perspective, and may have fewer opportunities for peer support (i.e., having contemporaries with whom they can share their common experience of having had cancer). Conversely older survivors have more life experience, which might lead to better coping strategies, and they may face fewer work-related and social demands. This is supported by the findings from our study that younger survivors are more likely than older survivors to rate issues related to having children, financial difficulties, loss of future life plans, and lack of support as being relevant to them. Although some of these issues appear to be more relevant to younger survivors, in the interest of parsimony (i.e., not having to create two versions of a core survivorship questionnaire), we have decided to include them in our consolidated issue list.

A strength of our study is that we included a relatively large number of survivors from 11 cancer diagnosis groups from a total of 14 European countries. This enhances the generalizability of our findings. Also, our strategy with site-specific survivor modules ensures that relevant chronic physical symptoms are included. Furthermore, the retention of the items and scales of the QLQ-C30 that are still relevant for disease-free survivors will ensure continuity in the evaluation of HRQOL over time, from diagnosis through the long-term survivorship phase.

A possible limitation of our work is that those cancer survivors in our sample who were more than 5 years post-treatment were drawn primarily from hospital registries. Many patients who are 5 years or longer post-treatment may no longer be in active follow-up, and those who are may be those with more serious, chronic health problems. This could cause some degree of overestimation of the relevance of various survivorship issues in this subgroup of longer term survivors. Also, our sample was somewhat younger than one might expect based on the median age of the general population at cancer diagnosis (66 years) [47].

## Conclusions

We identified 116 generic survivorship issues, and on average, 26 site-specific survivorship issues per tumor site. Compared to existing cancer survivorship questionnaires, our findings underscore the relevance of assessing issues related to chronic physical side effects of treatment such as neuropathy and joint pain in addition to the psychosocial aspects of survivorship.

In the next phase of this project, we will further develop and test the core survivorship questionnaire, and we will also develop survivorship modules for breast, prostate, and colorectal cancer survivors. The choice of these three disease sites was based on the incidence, survival rates, and the number of survivorship studies conducted in these disease sites. In the longer term, we intend to develop survivorship modules for a much broader set of cancer sites. Ultimately, this will yield a comprehensive suite of survivorship questionnaires that will yield both a common data set for comparison of results across tumor sites, and unique information about the survivorship experience of specific groups of cancer survivors.

## Additional file

Additional file 1: The list of 134 articles included in the review. (PDF 118 kb)

## Abbreviations

CPILS: Cancer Problems in Living Scale
EORTC: European Organisation for Research and Treatment of Cancer
FACT-G: Functional Assessment of Cancer Therapy – General
HRQOL: Health-related quality of life
IOC/IOCv2: Impact of Cancer
MOS: Medical Outcomes study
N: Number
PROMs: Patient-reported outcome measures
QLACS: Quality of Life in Adult Cancer Survivors
QLG: Quality of Life Group
QLQ: Quality of life questionnaire
QLQ-C30: EORTC core questionnaire
QOL: Quality of life
QoL-CS: Quality of Life Cancer Survivors
SD: Standard deviation
SLDC-C: Satisfaction with Life Domains Scale for Cancer
TST: Time-since-completion-of-last-treatment
WHO: World Health Organization
