# Supplementary material for: Understanding the quality of life (QOL) issues in survivors of cancer: towards the development of an EORTC QOL cancer survivorship questionnaire
Source: Health Qual Life Outcomes. 2018 Jun 4;16:114. doi: 10.1186/s12955-018-0920-0 (PMC5987570; doi:10.1186/s12955-018-0920-0)
Supplement: Supplementary file 1 — The list of 134 articles included in the review. (PDF 118 kb) [file 12955_2018_920_MOESM1_ESM.pdf]

1. Ahmed RL, Prizment A, Lazovich D, Schmitz KH, Folsom AR: **Lymphedema and quality of life in breast cancer survivors: the Iowa Women's Health Study.** *J Clin Oncol* 2008, **26:**5689-5696.
2. Alfano CM, Lichstein KL, Vander Wal GS, Smith AW, Reeve BB, McTiernan A, Bernstein L, Baumgartner KB, Ballard-Barbash R: **Sleep duration change across breast cancer survivorship: associations with symptoms and health-related quality of life.** *Breast Cancer Res Treat* 2011, **130:**243-254.
3. Alfano CM, McGregor BA, Kuniyuki A, Reeve BB, Bowen DJ, Baumgartner KB, Bernstein L, Ballard-Barbash R, Malone KE, Ganz PA, McTiernan A: **Psychometric properties of a tool for measuring hormone-related symptoms in breast cancer survivors.** *Psychooncology* 2006, **15:**985-1000.
4. Alfano CM, McGregor BA, Kuniyuki A, Reeve BB, Bowen DJ, Wilder Smith A, Baumgartner KB, Bernstein L, Ballard-Barbash R, Malone KE, et al: **Psychometric evaluation of the Brief Cancer Impact Assessment among breast cancer survivors.** *Oncology* 2006, **70:**190-202.
5. Andersen BL, Woods XA, Copeland LJ: **Sexual self-schema and sexual morbidity among gynecologic cancer survivors.** *J Consult Clin Psychol* 1997, **65:**221-229.
6. Appleton L, Goodlad S, Irvine F, Poole H, Wall C: **Patients' experiences of living beyond colorectal cancer: a qualitative study.** *Eur J Oncol Nurs* 2013, **17:**610-617.
7. Arden-Close E, Absolom K, Greenfield DM, Hancock BW, Coleman RE, Eiser C: **Gender differences in self-reported late effects, quality of life and satisfaction with clinic in survivors of lymphoma.** *Psychooncology* 2011, **20:**1202-1210.
8. Ashing-Giwa KT, Lim JW: **Exploring the association between functional strain and emotional well-being among a population-based sample of breast cancer survivors.** *Psychooncology* 2010, **19:**150-159.
9. Ashing-Giwa KT, Lim JW: **Examining emotional outcomes among a multiethnic cohort of breast cancer survivors.** *Oncol Nurs Forum* 2011, **38:**279-288.
10. Ashing-Giwa KT, Padilla G, Tejero J, Kraemer J, Wright K, Coscarelli A, Clayton S, Williams I, Hills D: **Understanding the breast cancer experience of women: a qualitative study of African American, Asian American, Latina and Caucasian cancer survivors.** *Psychooncology* 2004, **13:**408-428.
11. Assing Hvidt E, Raun Iversen H, Ploug Hansen H: **'Someone to hold the hand over me': the significance of transpersonal 'attachment' relationships of Danish cancer survivors.** *Eur J Cancer Care (Engl)* 2013, **22:**726-737.
12. Avis NE, Smith KW, McGraw S, Smith RG, Petronis VM, Carver CS: **Assessing quality of life in adult cancer survivors (QLACS).** *Qual Life Res* 2005, **14:**1007-1023.
13. Azuero A, Su X, McNees P, Meneses K: **A revision of the quality of life-breast cancer survivors (QOL-BCS) instrument.** *Res Nurs Health* 2013, **36:**423-434.
14. Baker KS, Ness KK, Weisdorf D, Francisco L, Sun CL, Forman S, Bhatia S: **Late effects in survivors of acute leukemia treated with hematopoietic cell transplantation: a report from the Bone Marrow Transplant Survivor Study.** *Leukemia* 2010, **24:**2039-2047.
15. Ball M, Nelson CJ, Shuk E, Starr TD, Temple L, Jandorf L, Schover L, Mulhall JP, Woo H, Jennings S, DuHamel K: **Men's experience with sexual dysfunction post-rectal cancer**

**treatment: a qualitative study.** *J Cancer Educ* 2013, **28:**494-502.
16. Beesley V, Janda M, Eakin E, Obermair A, Battistutta D: **Lymphedema after gynecological cancer treatment : prevalence, correlates, and supportive care needs.** *Cancer* 2007, **109:**2607-2614.
17. Bellizzi KM, Miller MF, Arora NK, Rowland JH: **Positive and negative life changes experienced by survivors of non-Hodgkin's lymphoma.** *Ann Behav Med* 2007, **34:**188-199.
18. Bennett JA, Cameron LD, Brown PM, Whitehead LC, Porter D, Ottaway-Parkes T, Robinson E: **Time since diagnosis as a predictor of symptoms, depression, cognition, social concerns, perceived benefits, and overall health in cancer survivors.** *Oncol Nurs Forum* 2010, **37:**331-338.
19. Bentzen AG, Balteskard L, Wanderas EH, Frykholm G, Wilsgaard T, Dahl O, Guren MG: **Impaired health-related quality of life after chemoradiotherapy for anal cancer: late effects in a national cohort of 128 survivors.** *Acta Oncol* 2013, **52:**736-744.
20. Biglia N, Cozzarella M, Cacciari F, Ponzone R, Roagna R, Maggiorotto F, Sismondi P: **Menopause after breast cancer: a survey on breast cancer survivors.** *Maturitas* 2003, **45:**29-38.
21. Bishop MM, Curbow BA, Springer SH, Lee JA, Wingard JR: **Comparison of lasting life changes after cancer and BMT: perspectives of long-term survivors and spouses.** *Psychooncology* 2011, **20:**926-934.
22. Bloom JR, Stewart SL, Chang S, Banks PJ: **Then and now: quality of life of young breast cancer survivors.** *Psychooncology* 2004, **13:**147-160.
23. Bloom JR, Stewart SL, Oakley-Girvan I, Banks PJ, Shema S: **Quality of life of younger breast cancer survivors: persistence of problems and sense of well-being.** *Psychooncology* 2012, **21:**655-665.
24. Boykoff N, Moieni M, Subramanian SK: **Confronting chemobrain: an in-depth look at survivors' reports of impact on work, social networks, and health care response.** *J Cancer Surviv* 2009, **3:**223-232.
25. Branvall E, Derolf AR, Johansson E, Hultcrantz M, Bergmark K, Bjorkholm M: **Self-reported fertility in long-term survivors of acute myeloid leukemia.** *Ann Hematol* 2014, **93:**1491-1498.
26. Brunet J, Sabiston CM, Burke S: **Surviving breast cancer: women's experiences with their changed bodies.** *Body Image* 2013, **10:**344-351.
27. Buki LP, Garces DM, Hinestrosa MC, Kogan L, Carrillo IY, French B: **Latina breast cancer survivors' lived experiences: diagnosis, treatment, and beyond.** *Cultur Divers Ethnic Minor Psychol* 2008, **14:**163-167.
28. Bulkley J, McMullen CK, Hornbrook MC, Grant M, Altschuler A, Wendel CS, Krouse RS: **Spiritual well-being in long-term colorectal cancer survivors with ostomies.** *Psychooncology* 2013, **22:**2513-2521.
29. Canada AL, Schover LR: **The psychosocial impact of interrupted childbearing in long-term female cancer survivors.** *Psychooncology* 2012, **21:**134-143.
30. Cappiello M, Cunningham RS, Knobf MT, Erdos D: **Breast cancer survivors: information and support after treatment.** *Clin Nurs Res* 2007, **16:**278-293; discussion 294-301.
31. Carter J, Raviv L, Applegarth L, Ford JS, Josephs L, Grill E, Sklar C, Sonoda Y, Baser RE,

Barakat RR: **A cross-sectional study of the psychosexual impact of cancer-related infertility in women: third-party reproductive assistance.** *J Cancer Surviv* 2010, **4:**236-246.
32. Champion VL, Ziner KW, Monahan PO, Stump TE, Cella D, Smith LG, Bell CJ, Von Ah D, Sledge GW: **Development and psychometric testing of a breast cancer survivor self-efficacy scale.** *Oncol Nurs Forum* 2013, **40:**E403-410.
33. Clemmens DA, Knafl K, Lev EL, McCorkle R: **Cervical cancer: patterns of long-term survival.** *Oncol Nurs Forum* 2008, **35:**897-903.
34. Connell S, Patterson C, Newman B: **A qualitative analysis of reproductive issues raised by young Australian women with breast cancer.** *Health Care Women Int* 2006, **27:**94-110.
35. Courrech Staal EF, van Sandick JW, van Tinteren H, Cats A, Aaronson NK: **Health-related quality of life in long-term esophageal cancer survivors after potentially curative treatment.** *J Thorac Cardiovasc Surg* 2010, **140:**777-783.
36. Crespi CM, Ganz PA, Petersen L, Castillo A, Caan B: **Refinement and psychometric evaluation of the impact of cancer scale.** *J Natl Cancer Inst* 2008, **100:**1530-1541.
37. De Boer AG, Genovesi PI, Sprangers MA, Van Sandick JW, Obertop H, Van Lanschot JJ: **Quality of life in long-term survivors after curative transhiatal oesophagectomy for oesophageal carcinoma.** *Br J Surg* 2000, **87:**1716-1721.
38. De Padova S, Rosti G, Scarpi E, Salvioni R, Amadori D, De Giorgi U: **Expectations of survivors, caregivers and healthcare providers for testicular cancer survivorship and quality of life.** *Tumori* 2011, **97:**367-373.
39. Deimling GT, Bowman KF, Sterns S, Wagner LJ, Kahana B: **Cancer-related health worries and psychological distress among older adult, long-term cancer survivors.** *Psychooncology* 2006, **15:**306-320.
40. Deimling GT, Sterns S, Bowman KF, Kahana B: **The health of older-adult, long-term cancer survivors.** *Cancer Nurs* 2005, **28:**415-424.
41. Deimling GT, Sterns S, Bowman KF, Kahana B: **Functioning and activity participation restrictions among older adult, long-term cancer survivors.** *Cancer Invest* 2007, **25:**106-116.
42. Dirksen SR: **Search for meaning in long-term cancer survivors.** *J Adv Nurs* 1995, **21:**628-633.
43. Dorval M, Maunsell E, Deschenes L, Brisson J: **Type of mastectomy and quality of life for long term breast carcinoma survivors.** *Cancer* 1998, **83:**2130-2138.
44. Dunberger G, Lindquist H, Waldenstrom AC, Nyberg T, Steineck G, Avall-Lundqvist E: **Lower limb lymphedema in gynecological cancer survivors--effect on daily life functioning.** *Support Care Cancer* 2013, **21:**3063-3070.
45. Durna EM, Crowe SM, Leader LR, Eden JA: **Quality of life of breast cancer survivors: the impact of hormonal replacement therapy.** *Climacteric* 2002, **5:**266-276.
46. Edman L, Larsen J, Hägglund H, Gardulf A: **Health-related quality of life, symptom distress and sense of coherence in adult survivors of allogeneic stem-cell transplantation.** *European Journal of Cancer Care* 2001, **10:**124-130.
47. Einstein MH, Rash JK, Chappell RJ, Swietlik JM, Hollenberg JP, Connor JP: **Quality of life in cervical cancer survivors: patient and provider perspectives on common**

**complications of cervical cancer and treatment.** *Gynecol Oncol* 2012, **125:**163-167.
48. Elliott J, Fallows A, Staetsky L, Smith PW, Foster CL, Maher EJ, Corner J: **The health and well-being of cancer survivors in the UK: findings from a population-based survey.** *Br J Cancer* 2011, **105 Suppl 1:**S11-20.
49. Fallbjork U, Salander P, Rasmussen BH: **From "no big deal" to "losing oneself": different meanings of mastectomy.** *Cancer Nurs* 2012, **35:**E41-48.
50. Ferrell BR, Dow KH: **Portraits of cancer survivorship: a glimpse through the lens of survivors' eyes.** *Cancer Pract* 1996, **4:**76-80.
51. Ferrell BR, Dow KH, Grant M: **Measurement of the quality of life in cancer survivors.** *Qual Life Res* 1995, **4:**523-531.
52. Flechl B, Ackerl M, Sax C, Dieckmann K, Crevenna R, Gaiger A, Widhalm G, Preusser M, Marosi C: **Neurocognitive and sociodemographic functioning of glioblastoma long-term survivors.** *J Neurooncol* 2012, **109:**331-339.
53. Fleer J, Sleijfer D, Hoekstra H, Tuinman M, Klip E, Hoekstra-Weebers J: **Objective and subjective predictors of cancer-related stress symptoms in testicular cancer survivors.** *Patient Educ Couns* 2006, **64:**142-150.
54. Foley KL, Farmer DF, Petronis VM, Smith RG, McGraw S, Smith K, Carver CS, Avis N: **A qualitative exploration of the cancer experience among long-term survivors: comparisons by cancer type, ethnicity, gender, and age.** *Psychooncology* 2006, **15:**248-258.
55. Fu OS, Crew KD, Jacobson JS, Greenlee H, Yu G, Campbell J, Ortiz Y, Hershman DL: **Ethnicity and persistent symptom burden in breast cancer survivors.** *J Cancer Surviv* 2009, **3:**241-250.
56. Gacci M, Saleh O, Cai T, Gore JL, D'Elia C, Minervini A, Masieri L, Giannessi C, Lanciotti M, Varca V, et al: **Quality of life in women undergoing urinary diversion for bladder cancer: results of a multicenter study among long-term disease-free survivors.** *Health Qual Life Outcomes* 2013, **11:**43.
57. Galvan N, Buki LP, Garces DM: **Suddenly, a carriage appears: social support needs of Latina breast cancer survivors.** *J Psychosoc Oncol* 2009, **27:**361-382.
58. Ganz PA, Desmond KA, Leedham B, Rowland JH, Meyerowitz BE, Belin TR: **Quality of life in long-term, disease-free survivors of breast cancer: a follow-up study.** *J Natl Cancer Inst* 2002, **94:**39-49.
59. Gaudine A, Sturge-Jacobs M, Kennedy M: **The experience of waiting and life during breast cancer follow-up.** *Res Theory Nurs Pract* 2003, **17:**153-168.
60. Gemmill R, Sun V, Ferrell B, Krouse RS, Grant M: **Going with the flow: quality-of-life outcomes of cancer survivors with urinary diversion.** *J Wound Ostomy Continence Nurs* 2010, **37:**65-72.
61. Gotay CC, Pagano IS: **Assessment of Survivor Concerns (ASC): a newly proposed brief questionnaire.** *Health Qual Life Outcomes* 2007, **5:**15.
62. Grant M, McMullen CK, Altschuler A, Mohler MJ, Hornbrook MC, Herrinton LJ, Wendel CS, Baldwin CM, Krouse RS: **Gender differences in quality of life among long-term colorectal cancer survivors with ostomies.** *Oncol Nurs Forum* 2011, **38:**587-596.
63. Grinyer A: **The late effects of mantle field radiotherapy: the information and support needs of women survivors of Hodgkin's disease.** *Eur J Oncol Nurs* 2010,

**14:**183-189.
64. Grover S, Hill-Kayser CE, Vachani C, Hampshire MK, DiLullo GA, Metz JM: **Patient reported late effects of gynecological cancer treatment.** *Gynecol Oncol* 2012, **124:**399-403.
65. Hauken MA, Larsen TM, Holsen I: **Meeting reality: young adult cancer survivors' experiences of reentering everyday life after cancer treatment.** *Cancer Nurs* 2013, **36:**E17-26.
66. Henningsohn L, Wijkstrom H, Steven K, Pedersen J, Ahlstrand C, Aus G, Kallestrup EB, Bergmark K, Onelov E, Steineck G: **Relative importance of sources of symptom-induced distress in urinary bladder cancer survivors.** *Eur Urol* 2003, **43:**651-662.
67. Hodgkinson K, Butow P, Hunt GE, Pendlebury S, Hobbs KM, Lo SK, Wain G: **The development and evaluation of a measure to assess cancer survivors' unmet supportive care needs: the CaSUN (Cancer Survivors' Unmet Needs measure).** *Psychooncology* 2007, **16:**796-804.
68. Hormes JM, Lytle LA, Gross CR, Ahmed RL, Troxel AB, Schmitz KH: **The body image and relationships scale: development and validation of a measure of body image in female breast cancer survivors.** *J Clin Oncol* 2008, **26:**1269-1274.
69. Jefford M, Karahalios E, Pollard A, Baravelli C, Carey M, Franklin J, Aranda S, Schofield P: **Survivorship issues following treatment completion--results from focus groups with Australian cancer survivors and health professionals.** *J Cancer Surviv* 2008, **2:**20-32.
70. Jim HS, Purnell JQ, Richardson SA, Golden-Kreutz D, Andersen BL: **Measuring meaning in life following cancer.** *Qual Life Res* 2006, **15:**1355-1371.
71. Joly F, Henry-Amar M, Arveux P, Reman O, Tanguy A, Peny AM, Lebailly P, Mace-Lesec'h J, Vie B, Genot JY, et al: **Late psychosocial sequelae in Hodgkin's disease survivors: a French population-based case-control study.** *J Clin Oncol* 1996, **14:**2444-2453.
72. Joly F, Heron JF, Kalusinski L, Bottet P, Brune D, Allouache N, Mace-Lesec'h J, Couette JE, Peny J, Henry-Amar M: **Quality of life in long-term survivors of testicular cancer: a population-based case-control study.** *J Clin Oncol* 2002, **20:**73-80.
73. Keating NL, Nørredam M, Landrum MB, Huskamp HA, Meara E: **Physical and Mental Health Status of Older Long-Term Cancer Survivors.** *Journal of the American Geriatrics Society* 2005, **53:**2145-2152.
74. Khan NF, Evans J, Rose PW: **A qualitative study of unmet needs and interactions with primary care among cancer survivors.** *Br J Cancer* 2011, **105 Suppl 1:**S46-51.
75. Kim C, McGlynn KA, McCorkle R, Zheng T, Erickson RL, Niebuhr DW, Ma S, Zhang Y, Bai Y, Dai L, et al: **Fertility among testicular cancer survivors: a case-control study in the U.S.** *J Cancer Surviv* 2010, **4:**266-273.
76. Lopez-Class M, Perret-Gentil M, Kreling B, Caicedo L, Mandelblatt J, Graves KD: **Quality of life among immigrant Latina breast cancer survivors: realities of culture and enhancing cancer care.** *J Cancer Educ* 2011, **26:**724-733.
77. Main DS, Nowels CT, Cavender TA, Etschmaier M, Steiner JF: **A qualitative study of work and work return in cancer survivors.** *Psychooncology* 2005, **14:**992-1004.
78. Maliski SL, Sarna L, Evangelista L, Padilla G: **The aftermath of lung cancer: balancing**

**the good and bad.** *Cancer Nurs* 2003, **26:**237-244.
79. McGrath P: **Positive outcomes for survivors of haematological malignancies from a spiritual perspective.** *Int J Nurs Pract* 2004, **10:**280-291.
80. Molassiotis A, Chan CW, Yam BM, Chan ES, Lam CS: **Life after cancer: adaptation issues faced by Chinese gynaecological cancer survivors in Hong Kong.** *Psychooncology* 2002, **11:**114-123.
81. Mols F, Thong MS, Vissers P, Nijsten T, van de Poll-Franse LV: **Socio-economic implications of cancer survivorship: results from the PROFILES registry.** *Eur J Cancer* 2012, **48:**2037-2042.
82. Mols F, Thong MS, Vreugdenhil G, van de Poll-Franse LV: **Long-term cancer survivors experience work changes after diagnosis: results of a population-based study.** *Psychooncology* 2009, **18:**1252-1260.
83. Mols F, Vingerhoets AJ, Coebergh JW, van de Poll-Franse LV: **Well-being, posttraumatic growth and benefit finding in long-term breast cancer survivors.** *Psychol Health* 2009, **24:**583-595.
84. Mosher CE, DuHamel KN, Rini C, Corner G, Lam J, Redd WH: **Quality of life concerns and depression among hematopoietic stem cell transplant survivors.** *Support Care Cancer* 2011, **19:**1357-1365.
85. Nachreiner NM, Dagher RK, McGovern PM, Baker BA, Alexander BH, Gerberich SG: **Successful return to work for cancer survivors.** *Aaohn j* 2007, **55:**290-295.
86. Ness S, Kokal J, Fee-Schroeder K, Novotny P, Satele D, Barton D: **Concerns across the survivorship trajectory: results from a survey of cancer survivors.** *Oncol Nurs Forum* 2013, **40:**35-42.
87. Noorda EM, van Kreij RH, Vrouenraets BC, Nieweg OE, Muller M, Kroon BB, Aaronson NK: **The health-related quality of life of long-term survivors of melanoma treated with isolated limb perfusion.** *Eur J Surg Oncol* 2007, **33:**776-782.
88. Oliveria SA, Shuk E, Hay JL, Heneghan M, Goulart JM, Panageas K, Geller AC, Halpern AC: **Melanoma survivors: health behaviors, surveillance, psychosocial factors, and family concerns.** *Psychooncology* 2013, **22:**106-116.
89. Paskett ED, Herndon JE, 2nd, Day JM, Stark NN, Winer EP, Grubbs SS, Pavy MD, Shapiro CL, List MA, Hensley ML, et al: **Applying a conceptual model for examining health-related quality of life in long-term breast cancer survivors: CALGB study 79804.** *Psychooncology* 2008, **17:**1108-1120.
90. Pedersen AF, Rossen P, Olesen F, von der Maase H, Vedsted P: **Fear of recurrence and causal attributions in long-term survivors of testicular cancer.** *Psychooncology* 2012, **21:**1222-1228.
91. Phipps E, Braitman LE, Stites S, Leighton JC: **Quality of life and symptom attribution in long-term colon cancer survivors.** *J Eval Clin Pract* 2008, **14:**254-258.
92. Ploos van Amstel FK, van den Berg SW, van Laarhoven HW, Gielissen MF, Prins JB, Ottevanger PB: **Distress screening remains important during follow-up after primary breast cancer treatment.** *Support Care Cancer* 2013, **21:**2107-2115.
93. Ramanakumar AV, Balakrishna Y, Ramarao G: **Coping mechanisms among long-term survivors of breast and cervical cancers in Mumbai, India.** *Asian Pac J Cancer Prev* 2005, **6:**189-194.

94. Ramirez M, Altschuler A, McMullen C, Grant M, Hornbrook M, Krouse R: **"I didn't feel like I was a person anymore": realigning full adult personhood after ostomy surgery.** *Med Anthropol Q* 2014, **28:**242-259.
95. Ramirez M, McMullen C, Grant M, Altschuler A, Hornbrook MC, Krouse RS: **Figuring out sex in a reconfigured body: experiences of female colorectal cancer survivors with ostomies.** *Women Health* 2009, **49:**608-624.
96. Rasmussen DM, Elverdam B: **Cancer survivors' experience of time--Time disruption and time appropriation.** *Journal of Advanced Nursing* 2007, **57:**614-622.
97. Rasmussen DM, Elverdam B: **The meaning of work and working life after cancer: an interview study.** *Psychooncology* 2008, **17:**1232-1238.
98. Ridner SH, Bonner CM, Deng J, Sinclair VG: **Voices from the shadows: living with lymphedema.** *Cancer Nurs* 2012, **35:**E18-26.
99. Rosedale M, Fu MR: **Confronting the unexpected: temporal, situational, and attributive dimensions of distressing symptom experience for breast cancer survivors.** *Oncol Nurs Forum* 2010, **37:**E28-33.
100. Rossen PB, Pedersen AF, Zachariae R, von der Maase H: **Health-related quality of life in long-term survivors of testicular cancer.** *J Clin Oncol* 2009, **27:**5993-5999.
101. Rudberg L, Carlsson M, Nilsson S, Wikblad K: **Self-perceived physical, psychologic, and general symptoms in survivors of testicular cancer 3 to 13 years after treatment.** *Cancer Nurs* 2002, **25:**187-195.
102. Schlairet MC, Benton MJ: **Quality of life and perceived educational needs among older cancer survivors.** *J Cancer Educ* 2012, **27:**21-26.
103. Schneider EC, Malin JL, Kahn KL, Ko CY, Adams J, Epstein AM: **Surviving colorectal cancer : patient-reported symptoms 4 years after diagnosis.** *Cancer* 2007, **110:**2075-2082.
104. Schultz PN, Klein MJ, Beck ML, Stava C, Sellin RV: **Breast cancer: relationship between menopausal symptoms, physiologic health effects of cancer treatment and physical constraints on quality of life in long-term survivors.** *J Clin Nurs* 2005, **14:**204-211.
105. Sekse RJ, Gjengedal E, Raheim M: **Living in a changed female body after gynecological cancer.** *Health Care Women Int* 2013, **34:**14-33.
106. Shigaki CL, Madsen R, Wanchai A, Stewart BR, Armer JM: **Upper extremity lymphedema: presence and effect on functioning five years after breast cancer treatment.** *Rehabil Psychol* 2013, **58:**342-349.
107. Skaali T, Fossa SD, Bremnes R, Dahl O, Haaland CF, Hauge ER, Klepp O, Oldenburg J, Wist E, Dahl AA: **Fear of recurrence in long-term testicular cancer survivors.** *Psychooncology* 2009, **18:**580-588.
108. Smith AB, King M, Butow P, Luckett T, Grimison P, Toner GC, Stockler M, Hovey E, Stubbs J, Hruby G, et al: **The prevalence and correlates of supportive care needs in testicular cancer survivors: a cross-sectional study.** *Psychooncology* 2013, **22:**2557-2564.
109. So WK, Chan CW, Choi KC, Wan RW, Mak SS, Chair SY: **Perceived unmet needs and health-related quality of life of Chinese cancer survivors at 1 year after treatment.** *Cancer Nurs* 2013, **36:**E23-32.
110. Stava C, Weiss LT, Vassilopoulou-Sellin R: **Health profiles of 814 very long-term breast cancer survivors.** *Clin Breast Cancer* 2006, **7:**228-236.

111. Stava CJ, Lopez A, Vassilopoulou-Sellin R: **Health profiles of younger and older breast cancer survivors.** *Cancer* 2006, **107:**1752-1759.
112. Steiner JF, Cavender TA, Nowels CT, Beaty BL, Bradley CJ, Fairclough DL, Main DS: **The impact of physical and psychosocial factors on work characteristics after cancer.** *Psychooncology* 2008, **17:**138-147.
113. Sun V, Grant M, McMullen CK, Altschuler A, Mohler MJ, Hornbrook MC, Herrinton LJ, Baldwin CM, Krouse RS: **Surviving colorectal cancer: long-term, persistent ostomy-specific concerns and adaptations.** *J Wound Ostomy Continence Nurs* 2013, **40:**61-72.
114. Sweeney C, Schmitz KH, Lazovich D, Virnig BA, Wallace RB, Folsom AR: **Functional limitations in elderly female cancer survivors.** *J Natl Cancer Inst* 2006, **98:**521-529.
115. Syme ML, Delaney E, Wachen JS, Gosian J, Moye J: **Sexual self-esteem and psychosocial functioning in military veterans after cancer.** *J Psychosoc Oncol* 2013, **31:**1-12.
116. Tan FL, Loh SY, Su TT, Veloo VW, Ng LL: **Return to work in multi-ethnic breast cancer survivors--a qualitative inquiry.** *Asian Pac J Cancer Prev* 2012, **13:**5791-5797.
117. Taylor TR, Huntley ED, Sween J, Makambi K, Mellman TA, Williams CD, Carter-Nolan P, Frederick W: **An exploratory analysis of fear of recurrence among African-American breast cancer survivors.** *Int J Behav Med* 2012, **19:**280-287.
118. Tong MC, Lee KY, Yuen MT, Lo PS: **Perceptions and experiences of post-irradiation swallowing difficulties in nasopharyngeal cancer survivors.** *Eur J Cancer Care (Engl)* 2011, **20:**170-178.
119. Tsuchiya M, Horn S, Ingham R: **Development of the Psycho-social Discomfort Scale (PsDS): investigation of psychometric properties among Japanese breast cancer survivors.** *Psychooncology* 2012, **21:**161-167.
120. Urbaniec OA, Collins K, Denson LA, Whitford HS: **Gynecological cancer survivors: assessment of psychological distress and unmet supportive care needs.** *J Psychosoc Oncol* 2011, **29:**534-551.
121. Vaz AF, Conde DM, Costa-Paiva L, Morais SS, Esteves SB, Pinto-Neto AM: **Quality of life and adverse events after radiotherapy in gynecologic cancer survivors: a cohort study.** *Arch Gynecol Obstet* 2011, **284:**1523-1531.
122. Vaz AF, Pinto-Neto AM, Conde DM, Costa-Paiva L, Morais SS, Pedro AO, Esteves SB: **Quality of life and menopausal and sexual symptoms in gynecologic cancer survivors: a cohort study.** *Menopause* 2011, **18:**662-669.
123. Velthuis MJ, Peeters PH, Gijsen BC, van den Berg JP, Koppejan-Rensenbrink RA, Vlaeyen JW, May AM: **Role of fear of movement in cancer survivors participating in a rehabilitation program: a longitudinal cohort study.** *Arch Phys Med Rehabil* 2012, **93:**332-338.
124. Vistad I, Cvancarova M, Fossa SD, Kristensen GB: **Postradiotherapy morbidity in long-term survivors after locally advanced cervical cancer: how well do physicians' assessments agree with those of their patients?** *Int J Radiat Oncol Biol Phys* 2008, **71:**1335-1342.
125. Vistad I, Cvancarova M, Kristensen GB, Fossa SD: **A study of chronic pelvic pain after radiotherapy in survivors of locally advanced cervical cancer.** *J Cancer Surviv* 2011,

**5:**208-216.
126. Vistad I, Fossa SD, Kristensen GB, Dahl AA: **Chronic fatigue and its correlates in long-term survivors of cervical cancer treated with radiotherapy.** *Bjog* 2007, **114:**1150-1158.
127. Von Ah D, Habermann B, Carpenter JS, Schneider BL: **Impact of perceived cognitive impairment in breast cancer survivors.** *Eur J Oncol Nurs* 2013, **17:**236-241.
128. Wallwork L, Richardson A: **Beyond cancer: changes, problems and needs expressed by adult lymphoma survivors attending an out-patients clinic.** *Eur J Cancer Care (Engl)* 1994, **3:**122-132.
129. Wyatt G, Friedman LL: **Long-term female cancer survivors: quality of life issues and clinical implications.** *Cancer Nurs* 1996, **19:**1-7.
130. Wyatt G, Kurtz ME, Friedman LL, Given B, Given CW: **Preliminary testing of the Long-Term Quality of Life (LTQL) instrument for female cancer survivors.** *J Nurs Meas* 1996, **4:**153-170.
131. Zebrack BJ, Ganz PA, Bernaards CA, Petersen L, Abraham L: **Assessing the impact of cancer: development of a new instrument for long-term survivors.** *Psychooncology* 2006, **15:**407-421.
132. Zeng YC, Li D, Loke AY: **Life after cervical cancer: quality of life among Chinese women.** *Nurs Health Sci* 2011, **13:**296-302.
133. Zhao L, Portier K, Stein K, Baker F, Smith T: **Exploratory factor analysis of the Cancer Problems in Living Scale: a report from the American Cancer Society's Studies of Cancer Survivors.** *J Pain Symptom Manage* 2009, **37:**676-686.
134. Zietman AL, Sacco D, Skowronski U, Gomery P, Kaufman DS, Clark JA, Talcott JA, Shipley WU: **Organ conservation in invasive bladder cancer by transurethral resection, chemotherapy and radiation: results of a urodynamic and quality of life study on long-term survivors.** *J Urol* 2003, **170:**1772-1776.
